# Multiple Imputations Applied to the DREAM3 Phosphoproteomics Challenge: A Winning Strategy

**DOI:** 10.1371/journal.pone.0008012

**Published:** 2010-01-18

**Authors:** Nicolas Guex, Eugenia Migliavacca, Ioannis Xenarios

**Affiliations:** 1 Vital-IT group, Swiss Institute of Bioinformatics, Lausanne, Switzerland; 2 Bioinformatics Core Facility, Swiss Institute of Bioinformatics, Lausanne, Switzerland; Center for Genomic Regulation, Spain

## Abstract

DREAM is an initiative that allows researchers to assess how well their methods or approaches can describe and predict networks of interacting molecules [1]. Each year, recently acquired datasets are released to predictors ahead of publication. Researchers typically have about three months to predict the masked data or network of interactions, using any predictive method. Predictions are assessed prior to an annual conference where the best predictions are unveiled and discussed. Here we present the strategy we used to make a winning prediction for the DREAM3 phosphoproteomics challenge. We used Amelia II, a multiple imputation software method developed by Gary King, James Honaker and Matthew Blackwell[2] in the context of social sciences to predict the 476 out of 4624 measurements that had been masked for the challenge. To chose the best possible multiple imputation parameters to apply for the challenge, we evaluated how transforming the data and varying the imputation parameters affected the ability to predict additionally masked data. We discuss the accuracy of our findings and show that multiple imputations applied to this dataset is a powerful method to accurately estimate the missing data. We postulate that multiple imputations methods might become an integral part of experimental design as a mean to achieve cost savings in experimental design or to increase the quantity of samples that could be handled for a given cost.

## Introduction

**Figure 1:**
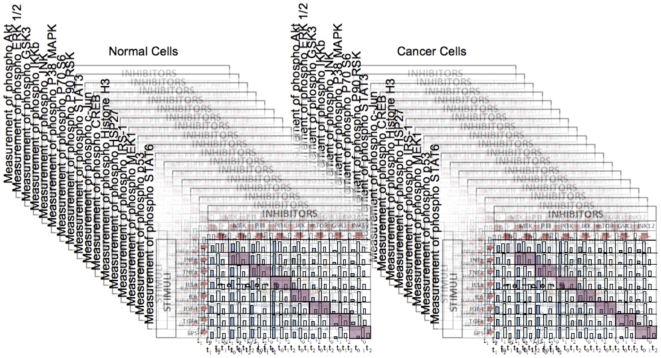
Description of the DREAM3 phosphoproteomics challenge. 17 phosphoproteins have been measured in Normal and Cancer Cells, following various combinations of Stimulus and Inhibitor at various time-points. A series of measurements (476 out of 4624) have been masked (diagonal). The challenge consisted in providing the most accurate prediction of those missing data.

DREAM is an initiative that is quite essential in the field of methods development to critically evaluate current computational methodologies (http://wiki.c2b2.columbia.edu/dream/index.php/The_DREAM_Project). In this respect, it follows the well-established Critical Assessment of methods of protein Structure Prediction (CASP) [3], [4], [5], [6], [7], [8], which has spurred innovation in this field. DREAM is now at its 4^th^ instance, and there is no doubt that it will become as beneficial for the Systems Biology world as CASP already is for the structural biology domain. We participated in the 3^rd^ instance of the DREAM challenge, in the phosphoproteomics section. Briefly, this challenge is based on a data set provided by Peter Sorger *et al* [9], where the authors measured the difference in signaling between normal and cancerous cells using phosphoproteomics assays. Predictors were given only 90% of the data and had to predict the value of the remaining measurements, which had been masked by the authors. This consisted in predicting the concentration of 17 phosphoproteins at two time points for 7 combinations of stimuli and inhibitors applied to normal and cancer hepatocytes (**Figure 1**). For each of the 17 phosphoproteins, 42 distinct combinations of stimuli and inhibitors measurements were given, in addition to un-stimulated and un-inhibited controls.

In this article, we describe the approach we took to analyze the data and make a winning prediction, and discuss the applicability of the process to other data sets. Given the complexity of the biological networks affected by the various stimuli and inhibitors, we decided to approach this challenge by imputing the missing data based solely on the exiting measured data. We took advantage of the Vital-IT high-performance computing center to run thousands of simulations to determine the best multiple imputation parameters to apply for our final prediction. This article will describe our approach in details. It is important to mention that, although our multiple imputations strategy resulted in a winning contribution, it does not provide any insights into the biomolecular system underlying the data. In other words, it does not infer nor uses the wiring structure of the signaling network. As a consequence, it would not be possible to infer the outcome of multiple simultaneous perturbations on the phosphoproteomics measurements using this approach. To this end, other methods that implicitly take advantage of the signaling network using kinetic modeling or logical modeling should be used [10]. These methods will likely be used in the 2009 DREAM challenges, as several groups are focusing their attention towards methodologies to infer and reconstruct regulatory networks and evaluate their dynamical behaviour.

## Analysis

**Figure 2:**
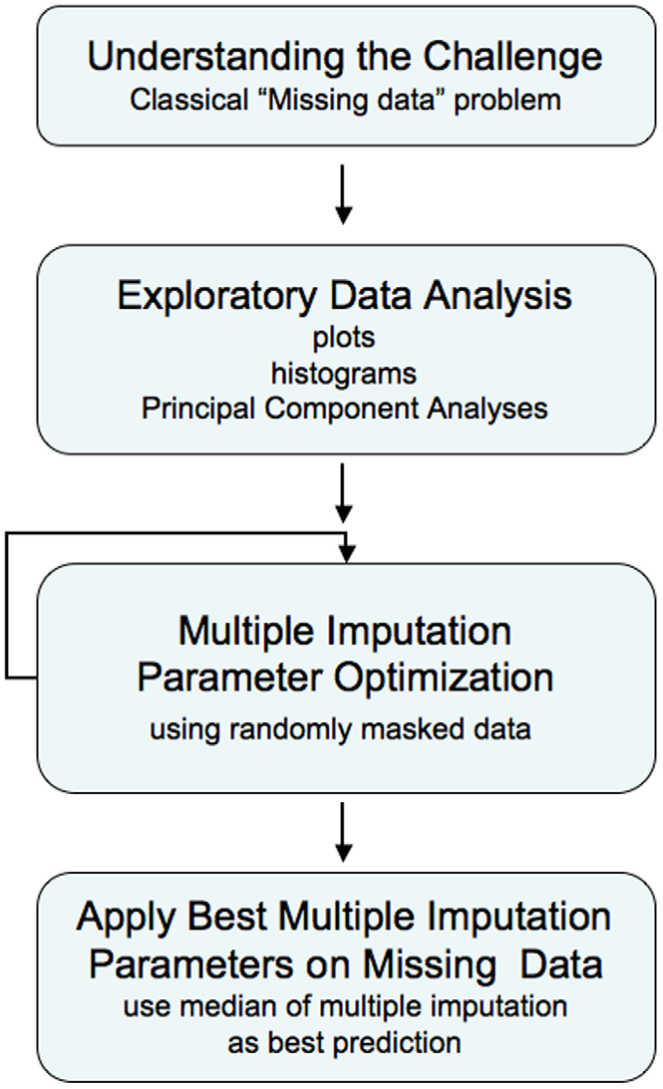
Analysis workflow summary. Description of the different steps applied to the DREAM challenge.

One interesting aspect of the DREAM challenge is that there is only about three months between the time the data are released and the due date for the analysis. This does not leave much time to develop and validate novel methods, and predictors typically apply methods they have been developing in their laboratory over time. We took a slightly different approach, which consisted in analyzing the problem, identifying a suitable tool to perform the analysis, tuning the parameters during the time allowed and performing our final prediction. The summary of the analysis workflow is described in **Figure 2**. Each step is described in more depth in the following sections.

### Step 1: Understanding the Challenge

We immediately recognized that the masked data could be assimilated to missing data. Missing data is a recurrent and very annoying problem, as most statistical tools do not tolerate missing data. Common ways to deal with this issue include ignoring samples as soon as one measurement is missing, which prunes the dataset. Although applicable in cases of large datasets with few missing values, this is far from ideal and inapplicable in our case, as it is indeed the objective of the challenge to predict the masked data. The other common approach is to replace the missing data either with random values, or by the mean or median of non-missing values. Both approaches can lead to biases and inefficiencies. Fortunately, solutions to impute the missing data have been developed, in particular in the field of social sciences, where multiple questions polls are usually only partially filled and where removing any sample partially filled would amount to discarding most of the dataset. We elected to use the Amelia II package[2] of R[11], a multiple imputation method described in depth in a report entitled “What to do About Missing Values in Time Series Cross-Section Data”, available at http://gking.harvard.edu/amelia/.

### Step 2: Performing Exploratory Data Analysis

**Figure 3:**
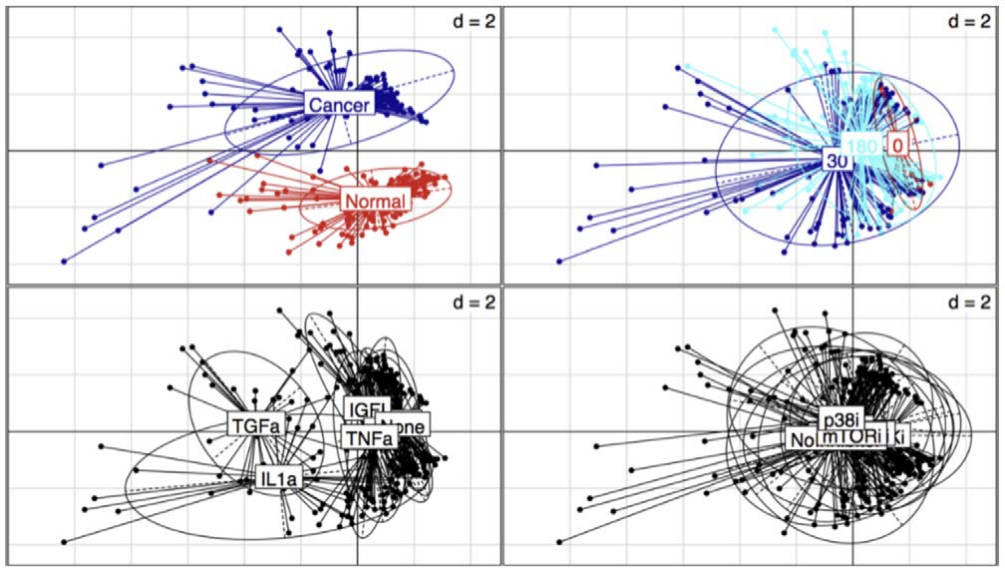
Inspection of the challenge data through Principal Component Analysis (PCA). All measurements classes were pooled together, irrespective of the CellType, Time, Stimulus and Inhibitor. Scatter plots with representation of the various classes were produced with the s.class command of the ade4 R package. The various classes are: Top left: CellType (Normal, Cancer). Top right: Time (0, 30, 180 mn). Bottom left: The seven stimuli. Bottom right: The seven inhibitors.

To get a “feel” for the data, we performed a principal component analysis (PCA) using the dudi.pca module of the ade4 package[12] of R (**Figure 3**). It is obviously apparent that there is a large difference between Cancer and Normal cells. Likewise, some grouping is also apparent for the various time points. Measurements at time zero and 180mn cluster in relatively tight neighboring regions of the PCA space. In contrast, there is a large dispersion of the measurements for time 30 mn. Moreover, those measurements tend to be further away from measurements at time zero than measurements at time 180 mn are. Those observations led us to try various parameters that would account for time effect and cell type effect (cross-section Normal *vs* Cancer) during the multiple imputation process.

### Step 3: Optimizing the Multiple Imputation Parameters

**Figure 4:**
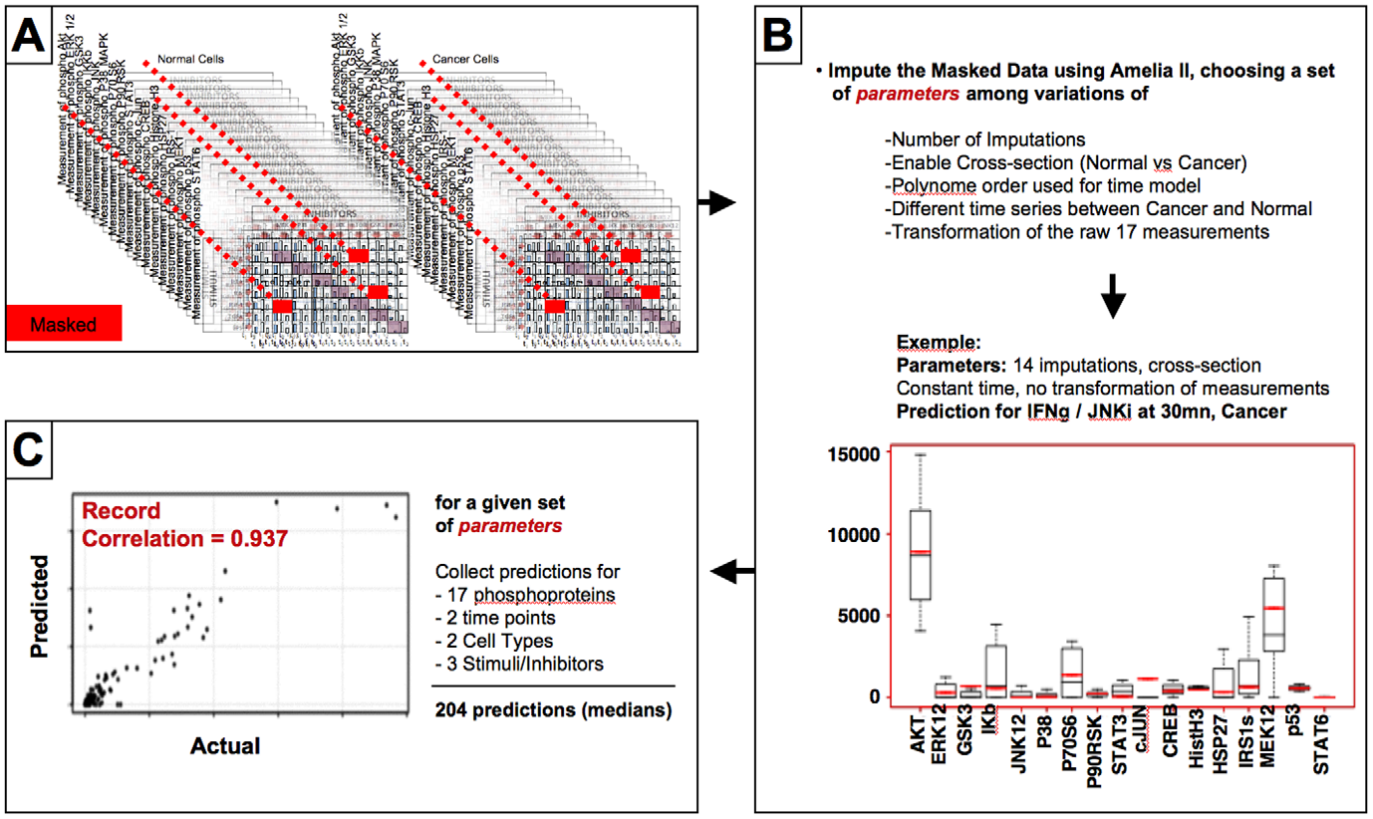
Identification of the best multiple imputation parameters. **A**. Selection of three Stimulus/Inhibitor pairs and masking (red) of the 17 associated phosphoproteomics measurements at 30 and 180 mn in both normal and cancer cells (17×3×2×2 = 204 masked measurements). **B**. Example of multiple imputations results with a given set of Amelia II parameters for the 17 masked phosphoproteomics measurements associated with an IFNγ stimulation and JNK inhibition at 30 mn in cancer cells. The boxplots show the spread of the 14 multiple imputations performed for each phophoprotein and the median of the prediction (black) can be compared to the actual measurement (red). **C**. The correlation between the median of each of the 204 predictions and the 204 actual measureents which have been masked is computed and provides an evaluation of the prediction performance for a given set of Amelia II input parameters.

Although any additional prior data already present in the literature could be used to help solve the challenge, we decided to use only the rich dataset at our disposition to make our predictions, since the conditions, laboratories and experimentalists affect experimental readouts. Therefore we committed to two principles before starting the analysis: (1) let the data drive the prediction process and (2) do not correct our predictions based on any particular biological knowledge. Amelia II has several input parameters, and can apply various transformations to the input data. To determine the best combination of parameters to use to impute the missing data of the challenge dataset, we randomly chose three Stimuli/Inhibitors pairs among the 42 combinations of Stimuli/Inhibitors for which we had data, with the restriction that a given Stimulus or Inhibitor could not be picked more than once. We then masked the 17 phosphoproteomics measurement data associated with those three pairs at time points 30 and 180 mn for Cancer and Normal cells. This corresponded to the masking of 204 (17×3×2×2) measurements. We then used Amelia II to impute the 204 masked data with various input parameters and assessed the performance of the prediction by computing the Pearson correlation coefficient between the median of multiple imputations and the actual measurement (**Figure 4**). The process was repeated 50 times, selecting different combinations of masked Stimulus/Inhibitors pairs. Thus, we collected 50 correlation coefficients for any set of multiple imputations parameters tested. To make our prediction for the challenge, we chose the set of parameters for which the median of the 50 Pearson Correlation coefficients was the highest. We then applied those parameters to the 476 masked data of the challenge.

As can be seen in **Figures S1 and S2**, the variation of the multiple imputations parameters influenced the ability to predict the masked data. In particular, increasing the number of multiple imputations improved the correlation (**Figure S1**). Likewise, increasing the polynomial order used to model the time effect was beneficial (vectors are parallels in the PCA space; **Figure S2**). Actually, we determined that the best correlation was achieved using a second order polynomial (data not shown). This is consistent with the observation that time points zero and 180 mn were close in the PCA space, whereas measurements belonging to the 30 mn time point were more distant and scattered (**Figure 3**). We also observed that when the cell status (Cancer, Normal) was considered as a cross-section, it was absolutely necessary to allow modeling of the time effect differently for each cell type. On another hand, the method used to initialize the imputation process (listwise deletion or identity matrix) had no effect. The best overall correlation (0.94) was obtained with 50 multiple imputations, a cross-section on the cell type and the possibility to apply a different model of the time effect for each cell type using a second order polynomial on the raw (untransformed) data.

## Discussion

When the number of imputations was large, we did not observe a statistical difference between imputing the missing data using untransformed or squared root transformed measurements, although we noticed a slightly tighter variance when untransformed data was used. Log transforming the data consistently gave inferior results (data not shown). However, we anticipated a beneficial effect of transforming the data, because during our initial data exploration phase, we observed that the measurements acquired for several of the 17 phosphoproteins were not normally distributed (data not shown). This violated the assumption made by the imputation model implemented in Amelia II, which optimally requires multivariate normally distributed data. During our search for optimal parameters, we either used the data as-is, or applied a squared root transformation on all measurements. As the various phosphoprotein measurements follow distinct distributions, we reasoned that the putative improvement obtained by transforming some measurements was compensated by the detrimental effect of transforming measurements that should have been left untransformed. Thus, we kept the multiple imputation parameters that gave us the best correlation with our own masked data and further evaluated the effect of transforming measurements for just some of the 17 phosphoproteins. We identified that a squared root transformation of Akt, IkBα, p38, p70S6 and HSP27 measurements modestly but significantly improved the overall correlation from 0.94 to 0.95 (unpaired t-test P-value 0.02). This is what we used for our final prediction.

**Figure 5:**
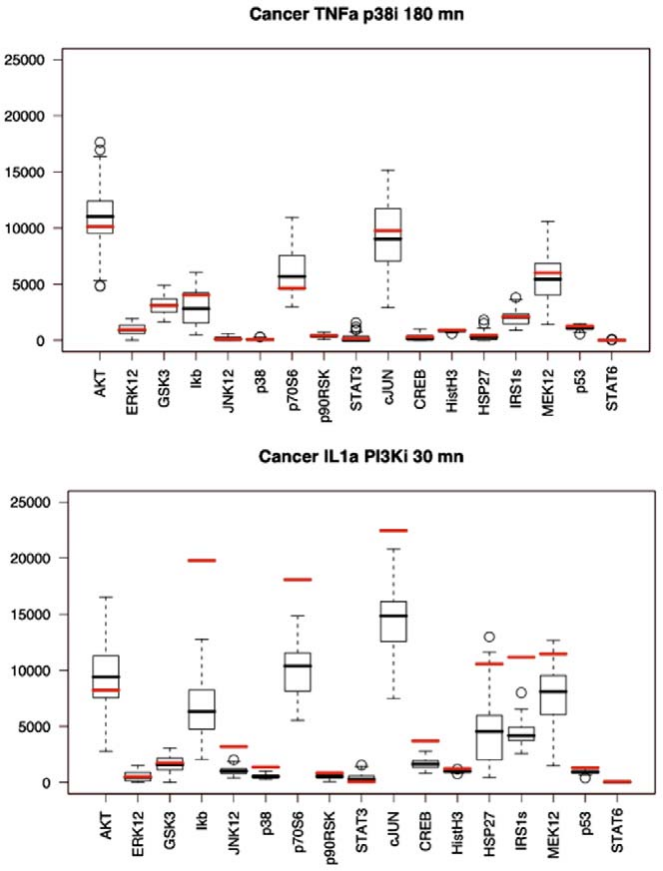
Evaluation of the quality of the DREAM3 challenge prediction. The multiple imputation process generated 50 predictions for each measurement, which are represented as boxplots. The median (black) was submitted as our prediction. In red, actual experimental measurement unveiled shortly before the DREAM3 conference. Top: example of high quality prediction. Bottom: Worse prediction.

Overall, the median of the multiple imputation process produced an extremely accurate estimation of the actual measured data. Representative predictions examples are provided in **Figure 5**. The jury evaluated the predictions using a normalized square error by comparing the predictions with a null-model in which the missing values were sampled from the dataset to estimate a p-value. In our case, the chance to obtain such a prediction randomly was of 10^−22^. The main advantage of using multiple imputations is that it naturally gives a prediction range for each missing value. We observed that the actual measurement fell out of this range for only 30 out of the 476 predictions, that is 6.3% of the time (**Table S1**). Interestingly, 14 of those “outliers” concern the combination of IL-1 stimulation with PI3K inhibition, and 10 (e.g. a third) are more specifically under-predicted for this specific combination of stimulus/inhibitor at 30 mn in cancer cells. The fact that a third of the “outliers” are found in this combination (out of the 28 distinct combinations of Stimulus/Inhibitor/CellType/Time for which the data had been masked) might reflect that PI3K inhibition can affect the apparent concentration of the IL-1 stimuli perceived by the cell. Indeed PI3K is linked in part with the rapid induction of IL-1R1[13]. The combination of TGFα stimulation with GSK3 inhibition also takes its share of outliers (4 out of 30), and there is evidence that both play an antagonizing role in the case of keratinocyte migration in HaCat cells, a cell type similar to the HepG2 cells used to produce the challenge data[14].

Interestingly, both IL-1 and TGFα stimuli clearly behave differently from the other stimuli in our preliminary PCA (**Figure 2**). Based on this observation, it was expected that it would be more challenging to accurately predict the missing values for those stimuli. To come with a more sensible prediction of IL-1, it might have been useful to benefit from results of other interleukin stimuli such as IL-8 or IL-6 to better cover the signaling space. The PCA (**Figure 2**) does not discriminate the various inhibitors, which appear superposed. This is consistent with the presence of biological cross-talks between those inhibitors, such as for example GSK3i and PI3Ki[15].

For the DREAM3 challenge, about 10.3% of the measurements had been masked. Once all of the actual measurements were made available, we masked 952 out of 4624 measurements (e.g. about 20.6% of the data) randomly drawn from time points other than zero. We then used the optimal prediction parameters determined earlier to predict the masked data. Here again, we observed that the multiple imputation process defined a range in which the actual measurement almost always fell. Indeed, the actual measurement fell out of this range for only 49 out of the 952 predictions, that is 5.1% of the time (**Table S2**). This time, no clear pattern of misprediction could be identified for the 49 “outliers”. This absence of clear pattern might be due to the fact that the masked data was missing completely at random in this case, which is the best situation for multiple imputations.

After the DREAM conference, out of curiosity, we also tested the multiple imputation method on another challenge dataset: The gene expression prediction challenge, whose dataset was generously provided by Neil Clarke *et al*. Briefly, the challenge consisted in predicting the expression level of 50 genes in a gat1Δ yeast strain, for different time points following the addition of an histidine synthesis inhibitor. The expression level of these 50 genes as well as 9285 others was provided for wild type and 3 other mutant strains. We first back transformed the data to obtain raw measurements from the log transformed data supplied, and formatted the data to place genes in rows and mutants in columns. Contrarily to the phosphoproteomics challenge, we did not attempt to identify the optimal multiple imputations parameters by predicting the measurements of additionally masked genes. We directly imputed the missing data using just one set of (arbitrary) parameters: cross-section on the various genes, modeling the effect of time with a 2^nd^ order polynomial not varying across the cross-section, and 100 multiple imputations. We then evaluated what would have been our performance using the evaluation scripts used by the assessors, which are available from http://wiki.c2b2.columbia.edu/dream/index.php/D3c3. Although the prediction might probably have been improved by careful tuning of the parameters, it turns out that with this simple protocol, we would already have achieved the 3^rd^ best prediction (**Table S3**), with a score significantly better than several other predictors. Unfortunately, we cannot comment on the merit and pitfalls of the various methods used by the participating teams, because only anonymous rankings are provided by the organizers, so as to encourage submissions of experimental methods. However, a thorough comparative study of the different submissions is under preparation: Robert J. Prill, Daniel Marbach, Julio Saez-Rodriguez, Gregoire Altan-Bonnet, Peter Sorger, Neil Clarke, Gustavo Stolovitzky, Lessons from the DREAM3 challenges (this title may change), DREAM3 collection, PLoS One (to be published).

From this work, we conclude that the multiple imputation method is a powerful technique that can be generally applied to many situations relevant to large-scale biological data acquisition where missing data are encountered, such as microarrays experiments [16]. This is also particularly relevant to longitudinal studies where patients might not come to every appointment, or where measurements might be missing for a variety of reasons. For example, in a longitudinal study examining 13 biomarkers as predictors of mortality, about 40% of the participants were missing information on one or more biomarker [17]. Although we applied multiple imputations to somewhat artificial conditions where known data are removed from a set, this work could be extended to influence the experimental design phase of new projects. Indeed, most of the current approaches rely on the use of checkerbox design (combinations of stimuli and inhibitor), which is very expensive both in time and in consumable price. Knowing that, for some datasets, as much as 20% of the data could be imputed could be used to reduce the amount of data to actually measure to reach a biological conclusion. This approach could also be used to plan a multi-step experiment approach in which the best combinations of stimuli and inhibitors worth measuring in the next experiment are “imputed” from the current experiment, reminiscent to the “pay as you go” strategy suggested for example in the protein-protein interactions field[18]. An other potential application could be to circumvent inherent limitations of some technologies. For example, flow cytometry cannot simultaneously quantify more than 10 cell surface markers. This is due to the difficulty to find fluorescently labeled antibodies whose emission spectra does not overlap, or to the lack of antibodies coupled to different fluorophores. It might be possible to design experiments where cells would be split in batches marked with near complete set of antibodies. For example, assuming that antibodies A and D cannot be used simultaneously, an experiment splitting cells into a first batch marked with antibodies A,B and C and a second batch marked with antibodies B,C and D, should make it possible to impute the missing measurements and thus obtain a prediction of markers A,B,C and D for each cell.

To conclude, we believe that initiatives such as DREAM and ENFIN[19], which both provide a framework where the predictive power of computational methods can be rigorously benchmarked against experimental data should be encouraged. The structural biology community benefited strongly from CASP, and the systems biology and reverse-engineering fields will without doubt benefit from such initiatives.

## Supporting Information

Figure S1 Overall effect of varying the multiple imputation parameters. The process presented in Figure 4 has been repeated 50 times, masking different selections of 3 pairs of Stimuli/Inhibitors. In each case, 32 distinct combinations of parameters were tested, with 18 distinct number of multiple imputations (1–10, 15, 20, 25, 30, 35, 40, 45 and 50). For each of those 576 (32x18) parameters (x axis), the distribution of the 50 correlations computed as described in Figure 4C is presented as a boxplot. It is immediately apparent that for any of the 32 combinations of parameters tested, increasing the number of multiple imputations improves the prediction accurracy, but reaches a plateau after about 40 multiple imputations.
(0.14 MB DOC)

Figure S2 Principal Component Analysis of the multiple imputation parameters effect. #imputations: number of multiple imputations. Sqrt: Effect of applying a squared root transformation on all input data. Polytime: Effect of increasing the polynome order used to model the effect of time. Cross-section: indicates whether we should consider the cell status (Cancer, Normal) as a cross-section. Model cross-section time indicates whether the effect of the time should be modeled differently for Cancer and Normal cells.
(0.06 MB DOC)

Table S1 List of the 30 combinations of Stimulus/Inhibition/timepoint/CellType measurements (out of 476) whose actual value falls outside of the min-max prediction range defined by the multiple imputation process.
(0.09 MB DOC)

Table S2 List of the 49 combinations of Stimulus/Inhibition/timepoint/CellType measurements (out of 952 measurements masked completely at random) whose actual value falls outside of the min-max prediction range defined by the multiple imputations process.
(0.13 MB DOC)

Table S3 Assessment of how the multiple imputation method would have performed on the DREAM3 Expression Challenge. Score: log-transformed “average” of the overall gene-profile p-value and the overall time-profile P-value, computed as -0.5 log10 (GeneProfile*TimeProfile); larger scores indicate greater statistical significance of the prediction. Overall Gene-Profile P-value: geometric mean of the 50 gene-profile P-values for a given time point. Overall Time-Profile P-value: geometric mean of the 8 time-profile p-values for a given gene. Assessment details can be found on the DREAM website at http://wiki.c2b2.columbia.edu/dream/results/DREAM3/?c=3_1
(0.04 MB DOC)
