# Supplementary figures and images for: Multiple Imputations Applied to the DREAM3 Phosphoproteomics Challenge: A Winning Strategy
Source: PLoS One. 2010 Jan 18;5(1):e8012. doi: 10.1371/journal.pone.0008012 (PMC2807461; doi:10.1371/journal.pone.0008012)

**Figure S1**


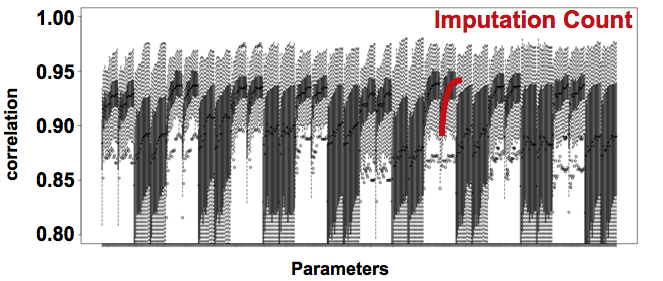

Supplement: Figure S1 — Overall effect of varying the multiple imputation parameters. The process presented in Figure 4 has been repeated 50 times, masking different selections of 3 pairs of Stimuli/Inhibitors. In each case, 32 distinct combinations of parameters were tested, with 18 distinct number of multiple imputations (1–10, 15, 20, 25, 30, 35, 40, 45 and 50). For each of those 576 (32x18) parameters (x axis), the distribution of the 50 correlations computed as described in Figure 4C is presented as a boxplot. It is immediately apparent that for any of the 32 combinations of parameters tested, increasing the number of multiple imputations improves the prediction accurracy, but reaches a plateau after about 40 multiple imputations. (0.14 MB DOC) [file pone.0008012.s001.doc]

**Figure S2**


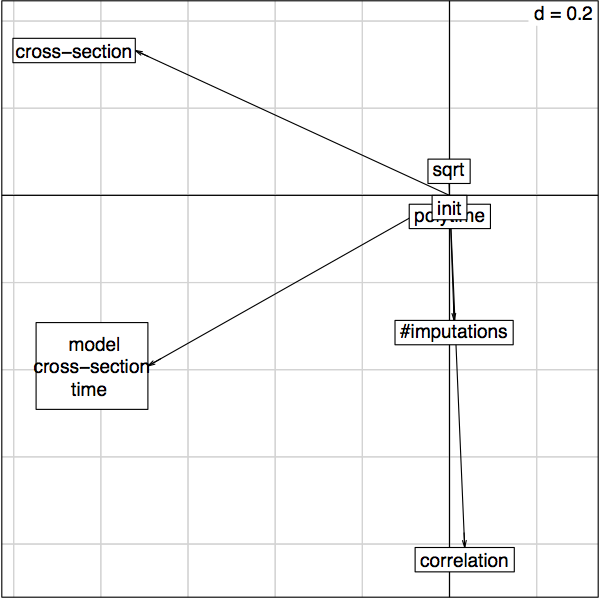

Supplement: Figure S2 — Principal Component Analysis of the multiple imputation parameters effect. #imputations: number of multiple imputations. Sqrt: Effect of applying a squared root transformation on all input data. Polytime: Effect of increasing the polynome order used to model the effect of time. Cross-section: indicates whether we should consider the cell status (Cancer, Normal) as a cross-section. Model cross-section time indicates whether the effect of the time should be modeled differently for Cancer and Normal cells. (0.06 MB DOC) [file pone.0008012.s002.doc]
