# Supplementary material for: Multiple Imputations Applied to the DREAM3 Phosphoproteomics Challenge: A Winning Strategy
Source: PLoS One. 2010 Jan 18;5(1):e8012. doi: 10.1371/journal.pone.0008012 (PMC2807461; doi:10.1371/journal.pone.0008012)
Supplement: Table S1 — List of the 30 combinations of Stimulus/Inhibition/timepoint/CellType measurements (out of 476) whose actual value falls outside of the min-max prediction range defined by the multiple imputation process. (0.09 MB DOC) [file pone.0008012.s003.doc]

Table S1

| **CellType** | **Stimulus** | **Inhibitor** | **Time (mn)** | **Phospho-protein** | **Measurement value** | **Median**  **Prediction** |  |
| --- | --- | --- | --- | --- | --- | --- | --- |
| Normal | IFNg | MEKi | 30 | AKT | 3632 | 3766 | over |
| Normal | TNFa | p38i | 30 | HSP27 | 2525 | 3021 | over |
| Normal | TNFa | p38i | 30 | STAT6 | 11 | 20 | over |
| Normal | IL6 | IKKi | 30 | STAT3 | 5375 | 4702 | under |
| Normal | IL1a | PI3Ki | 180 | Ikb | 1154 | 1705 | over |
| Normal | IL1a | PI3Ki | 180 | JNK12 | 195 | 292 | over |
| Normal | IL1a | PI3Ki | 180 | p38 | 334 | 472 | over |
| Normal | IL1a | PI3Ki | 180 | IRS1s | 1091 | 1387 | over |
| Normal | TGFa | GSK3i | 180 | ERK12 | 4276 | 4053 | under |
| Cancer | IFNg | MEKi | 30 | HistH3 | 1081 | 1078 | under |
| Cancer | IFNg | MEKi | 30 | MEK12 | 6448 | 8244 | over |
| Cancer | IL1a | PI3Ki | 30 | Ikb | 19782 | 12763 | under |
| Cancer | IL1a | PI3Ki | 30 | JNK12 | 3183 | 2013 | under |
| Cancer | IL1a | PI3Ki | 30 | p38 | 1347 | 994 | under |
| Cancer | IL1a | PI3Ki | 30 | p70S6 | 18068 | 14849 | under |
| Cancer | IL1a | PI3Ki | 30 | p90RSK | 835 | 831 | under |
| Cancer | IL1a | PI3Ki | 30 | cJUN | 22463 | 20809 | under |
| Cancer | IL1a | PI3Ki | 30 | CREB | 3681 | 2777 | under |
| Cancer | IL1a | PI3Ki | 30 | HistH3 | 1225 | 1179 | under |
| Cancer | IL1a | PI3Ki | 30 | IRS1s | 11172 | 8006 | under |
| Cancer | IL1a | PI3Ki | 30 | p53 | 1293 | 1253 | under |
| Cancer | IL6 | IKKi | 30 | STAT3 | 4919 | 4648 | under |
| Cancer | TGFa | GSK3i | 30 | ERK12 | 1066 | 1198 | over |
| Cancer | TGFa | GSK3i | 30 | CREB | 2783 | 2722 | under |
| Cancer | IL6 | IKKi | 180 | STAT3 | 1194 | 2019 | over |
| Cancer | IL6 | IKKi | 180 | p53 | 1884 | 1618 | under |
| Cancer | IGFI | mTORi | 180 | AKT | 23565 | 22435 | under |
| Cancer | IGFI | mTORi | 180 | GSK3 | 7905 | 5770 | under |
| Cancer | TGFa | GSK3i | 180 | cJUN | 21149 | 17473 | under |
| Cancer | LPS | JNKi | 180 | HistH3 | 290 | 355 | over |
