# Supplementary material for: Multiple Imputations Applied to the DREAM3 Phosphoproteomics Challenge: A Winning Strategy
Source: PLoS One. 2010 Jan 18;5(1):e8012. doi: 10.1371/journal.pone.0008012 (PMC2807461; doi:10.1371/journal.pone.0008012)
Supplement: Table S2 — List of the 49 combinations of Stimulus/Inhibition/timepoint/CellType measurements (out of 952 measurements masked completely at random) whose actual value falls outside of the min-max prediction range defined by the multiple imputations process. (0.13 MB DOC) [file pone.0008012.s004.doc]

Table S2

| **CellType** | **Stimulus** | **Inhibitor** | **Time (mn)** | **Phospho-protein** | **Measurement Value** | **Median Prediction** |  |
| --- | --- | --- | --- | --- | --- | --- | --- |
| Normal | IFNg | GSK3i | 30 | p70S6 | 4086 | 2595 | under |
| Normal | IGFI | NoInhibitor | 30 | GSK3 | 935 | 2609 | over |
| Normal | IGFI | mTORi | 30 | p70S6 | 1496 | 3412 | over |
| Normal | IL1a | NoInhibitor | 30 | Ikb | 1576 | 3588 | over |
| Normal | IL1a | NoInhibitor | 30 | GSK3 | 1627 | 2784 | over |
| Normal | IL6 | JNKi | 30 | STAT3 | 5191 | 3125 | under |
| Normal | IL6 | PI3Ki | 30 | STAT3 | 4221 | 3438 | under |
| Normal | IL6 | PI3Ki | 30 | STAT6 | 115 | 57 | under |
| Normal | LPS | MEKi | 30 | p70S6 | 8305 | 5094 | under |
| Normal | TGFa | MEKi | 30 | AKT | 20756 | 11621 | under |
| Normal | TGFa | NoInhibitor | 30 | MEK12 | 24543 | 19415 | under |
| Normal | TNFa | MEKi | 30 | AKT | 6671 | 12274 | over |
| Normal | TNFa | MEKi | 30 | MEK12 | 15436 | 11663 | under |
| Normal | IL6 | p38i | 180 | HSP27 | 1872 | 7286 | over |
| Normal | IL6 | JNKi | 180 | STAT3 | 1083 | 2979 | over |
| Normal | IL6 | PI3Ki | 180 | p90RSK | 178 | 391 | over |
| Normal | IL6 | NoInhibitor | 180 | STAT3 | 2264 | 3344 | over |
| Normal | LPS | mTORi | 180 | AKT | 8051 | 5761 | under |
| Normal | LPS | mTORi | 180 | HSP27 | 20824 | 11011 | under |
| Cancer | IGFI | JNKi | 30 | AKT | 23518 | 14906 | under |
| Cancer | IGFI | IKKi | 30 | IRS1s | 2573 | 1861 | under |
| Cancer | IL1a | IKKi | 30 | HSP27 | 7575 | 29258 | over |
| Cancer | IL1a | MEKi | 30 | ERK12 | 106 | 0 | under |
| Cancer | IL1a | mTORi | 30 | CREB | 3464 | 2692 | under |
| Cancer | IL1a | JNKi | 30 | CREB | 3435 | 2225 | under |
| Cancer | IL6 | MEKi | 30 | STAT6 | 6 | 61 | over |
| Cancer | IL6 | PI3Ki | 30 | STAT3 | 4461 | 2879 | under |
| Cancer | TGFa | NoInhibitor | 30 | MEK12 | 23367 | 16724 | under |
| Cancer | TGFa | MEKi | 30 | p90RSK | 161 | 947 | over |
| Cancer | TGFa | MEKi | 30 | ERK12 | 128 | 2451 | over |
| Cancer | TNFa | MEKi | 30 | ERK12 | 99 | 0 | under |
| Cancer | IFNg | GSK3i | 180 | HistH3 | 863 | 696 | under |
| Cancer | IGFI | mTORi | 180 | GSK3 | 7905 | 4619 | under |
| Cancer | IGFI | IKKi | 180 | IRS1s | 3082 | 2327 | under |
| Cancer | IGFI | JNKi | 180 | HistH3 | 328 | 625 | over |
| Cancer | IGFI | MEKi | 180 | GSK3 | 7336 | 4512 | under |
| Cancer | IGFI | JNKi | 180 | p53 | 524 | 828 | over |
| Cancer | IGFI | IKKi | 180 | GSK3 | 7974 | 5649 | under |
| Cancer | IGFI | mTORi | 180 | AKT | 23565 | 17222 | under |
| Cancer | IL1a | PI3Ki | 180 | p38 | 769 | 374 | under |
| Cancer | IL1a | JNKi | 180 | p38 | 880 | 416 | under |
| Cancer | IL1a | p38i | 180 | JNK12 | 259 | 761 | over |
| Cancer | IL1a | JNKi | 180 | Ikb | 8433 | 4549 | under |
| Cancer | IL1a | mTORi | 180 | cJUN | 21153 | 14916 | under |
| Cancer | IL6 | IKKi | 180 | STAT3 | 1194 | 3225 | over |
| Cancer | LPS | GSK3i | 180 | HSP27 | 227 | 1844 | over |
| Cancer | TGFa | MEKi | 180 | ERK12 | 519 | 3430 | over |
| Cancer | TGFa | GSK3i | 180 | cJUN | 21149 | 14141 | under |
| Cancer | TGFa | MEKi | 180 | p90RSK | 132 | 787 | over |
