# Supplementary material for: Multiple Imputations Applied to the DREAM3 Phosphoproteomics Challenge: A Winning Strategy
Source: PLoS One. 2010 Jan 18;5(1):e8012. doi: 10.1371/journal.pone.0008012 (PMC2807461; doi:10.1371/journal.pone.0008012)
Supplement: Table S3 — Assessment of how the multiple imputation method would have performed on the DREAM3 Expression Challenge. Score: log-transformed “average” of the overall gene-profile p-value and the overall time-profile P-value, computed as -0.5 log10 (GeneProfile*TimeProfile); larger scores indicate greater statistical significance of the prediction. Overall Gene-Profile P-value: geometric mean of the 50 gene-profile P-values for a given time point. Overall Time-Profile P-value: geometric mean of the 8 time-profile p-values for a given gene. Assessment details can be found on the DREAM website at http://wiki.c2b2.columbia.edu/dream/results/DREAM3/?c=3_1 (0.04 MB DOC) [file pone.0008012.s005.doc]

**Table S3**

| **Team** | **Score** | **Overall GeneProfile Pval** | **Overall TimeProfile Pval** |
| --- | --- | --- | --- |
| Team 301 | 3.252 | 6.511e-06 | 4.811e-02 |
| Team 287 | 3.183 | 1.114e-05 | 3.858e-02 |
| Multiple Imputations | 2.309 | 3.198e-04 | 7.550e-02 |
| Team 263 | 1.849 | 7.527e-04 | 2.661e-01 |
| Team 297 | 1.677 | 5.574e-03 | 7.932e-02 |
| Team 126 | 1.458 | 8.966e-03 | 1.354e-01 |
| Team 273 | 1.328 | 6.111e-03 | 3.620e-01 |
| Team 186 | 0.985 | 3.857e-02 | 2.773e-01 |
| Team 190 | 0.891 | 4.210e-02 | 3.921e-01 |
| Team 193 | 0.197 | 7.387e-01 | 5.473-e01 |
